# A survey of attitudes and factors associated with successful cardiopulmonary resuscitation (CPR) knowledge transfer in an older population most likely to witness cardiac arrest: design and methodology

**DOI:** 10.1186/1471-227X-8-13

**Published:** 2008-11-05

**Authors:** Christian Vaillancourt, Jeremy Grimshaw, Jamie C Brehaut, Martin Osmond, Manya L Charette, George A Wells, Ian G Stiell

**Affiliations:** 1 Ottawa Health Research Institute, Clinical Epidemiology Program, Ottawa, Canada; 2 Department of Emergency Medicine, University of Ottawa, Ottawa, Canada; 3 Department of Medicine, University of Ottawa, Ottawa, Canada; 4 Department of Epidemiology and Community Medicine, University of Ottawa, Ottawa, Canada; 5 Department of Pediatrics, University of Ottawa, Ottawa, Canada

## Abstract

**Background:**

Overall survival rates for out-of-hospital cardiac arrest rarely exceed 5%. While bystander cardiopulmonary resuscitation (CPR) can increase survival for cardiac arrest victims by up to four times, bystander CPR rates remain low in Canada (15%). Most cardiac arrest victims are men in their sixties, they usually collapse in their own home (85%) and the event is witnessed 50% of the time. These statistics would appear to support a strategy of targeted CPR training for an older population that is most likely to witness a cardiac arrest event. However, interest in CPR training appears to decrease with advancing age. Behaviour surrounding CPR training and performance has never been studied using well validated behavioural theories.

**Methods/Design:**

The overall goal of this study is to conduct a survey to better understand the behavioural factors influencing CPR training and performance in men and women 55 years of age and older. The study will proceed in three phases. In phase one, semi-structured qualitative interviews will be conducted and recorded to identify common categories and themes regarding seeking CPR training and providing CPR to a cardiac arrest victim. The themes identified in the first phase will be used in phase two to develop, pilot-test, and refine a survey instrument based upon the Theory of Planned Behaviour. In the third phase of the project, the final survey will be administered to a sample of the study population over the telephone. Analyses will include measures of sampling bias, reliability of the measures, construct validity, as well as multiple regression analyses to identify constructs and beliefs most salient to seniors' decisions about whether to attend CPR classes or perform CPR on a cardiac arrest victim.

**Discussion:**

The results of this survey will provide valuable insight into factors influencing the interest in CPR training and performance among a targeted group of individuals most susceptible to witnessing a victim in cardiac arrest. The findings can then be applied to the design of trials of various interventions designed to promote attendance at CPR classes and improve CPR performance.

**Trial registration:**

ClinicalTrials.gov NCT00665288

## Background

### Definition and epidemiology of out-of-hospital cardiac arrest

Cardiac arrest refers to the sudden cessation of cardiac mechanical activity as confirmed by the absence of signs of circulation [1]. The victim collapses when the cardiac mechanical activity becomes too limited to provide adequate blood flow and oxygen to the brain and muscles. The victim is perceived to be lifeless if no vital signs are detectable (responsiveness, pulse, respiration). Electrical cardiac activity (ventricular fibrillation [VF], ventricular tachycardia [VT], or pulseless electrical activity [PEA]) seen on a cardiac monitor may become the only sign of vital activity. In the absence of cardiopulmonary resuscitation (CPR) and/or electrical defibrillation, such electrical cardiac activity is followed by asystole and then by death in a matter of minutes.

Cardiac arrest remains the leading cause of mortality in North America. The incidence of out-of-hospital cardiac arrest in Canada is estimated to be 55 per 100,000 of population [2], resulting in more than 17,875 deaths annually. Coronary artery disease is the most frequent condition leading to cardiac arrest [3]. More than 40% of all deaths from heart disease occur suddenly, and often constitute the victim's first manifestation of heart disease [4]. Sixty-five percent of all cardiac arrests occur outside the hospital setting [5], where the overall rate of survival to hospital discharge rarely exceeds 5% [2]. Survivors have a quality of life (Health Utilities Index Mark 3) similar to the general population [6]. Most commonly, cardiac arrest victims are men in their sixties. Canadian cardiac arrest victims collapse in their own home 85% of the time and 50% are witnessed by a family member or bystander [2]. However, bystander CPR rates remain low in Canada, and rarely exceed 15% of all cases in Ontario [2].

### The Chain of Survival

The American Heart Association "Chain of Survival" illustrates important concepts in the community response to out-of-hospital cardiac arrest [7]. The chain metaphor implies that cardiac arrest care is only as strong as its weakest link among the four links in the chain: 1) Early access: When dialling 9-1-1, a caller is put in communication with personnel that will appropriately dispatch police, fire, emergency medical services (EMS), or all three. In the case of a medical emergency, the call will rapidly be transferred to a medical dispatch centre. Dispatch Officers collect information on the nature of the medical emergency and dispatch the appropriate EMS unit(s), often as more information is being collected. 2) Early CPR: CPR can be defined as a succession of lung insufflations and chest compressions performed by a rescuer with the intention of restoring spontaneous circulation. Although police, fire, and EMS paramedics have all been trained to perform CPR in cases of cardiac arrest, it is what occurs during the first minutes before their arrival that is most crucial to the victim. A victim is almost four times more likely to survive a cardiac arrest event when receiving citizen bystander CPR before emergency personnel arrives [OR 3.7 (95% CI 2.5–5.4)] [8]. 3) Early defibrillation: Defibrillation occurs when myocardial cells in a chaotic or abnormal electrical rhythm, VF or VT, are depolarized at the same time by the delivery of an electrical current. This results in the re-establishment of a rhythmic and organized heart beat. Defibrillation can only occur when the heart exhibits disorganized electrical activity and is never successful in the case of asystole or PEA. 4) Early Advanced Care: Advanced cardiac life support care is defined by the use of definitive airway management such as endotracheal intubation, intravenous access and administration of drugs. Such drugs serve the purpose of increasing the coronary perfusion pressure by increasing peripheral vascular resistance (Epinephrine, Vasopressin), or to promote arrhythmia termination either alone by acting on myocardial cell electric action potential and/or by facilitating defibrillation (Lidocaine, Procainamide, Amiodarone). This being said, early advanced care failed to improve survival to hospital discharge in the largest prospective prehospital study conducted to date [8].

### Low bystander CPR rates

Data from Seattle indicates that a survival rate of 30% can be achieved for witnessed VF cardiac arrest victims receiving bystander CPR [9]. Other communities such as Akita and Otsu, Japan report overall survival rates from cardiac arrest of 15% and 9%, respectively, in association with bystander CPR rates of 49% and 29% [10]. In comparison, citizen bystander CPR and overall survival rates are rather modest in most Canadian provinces, rarely exceeding 15% and 5% respectively [2].

Various attempts have been made to improve bystander CPR rates in the past. Two very popular and contrasting approaches are mass CPR training events and targeted CPR training of family members of patients suffering from cardiovascular disease. The objective of the mass CPR training is to teach as many CPR providers as possible from the general population in a group setting. Although mass CPR training events can reach groups of a few hundred to thousands of participants at a time [11-14], these events usually attract young participants unlikely to witness cardiac arrest. In addition, they are not cost-effective[15] and their effect on survival has not been demonstrated. Targeting the population at large may not achieve the desired goal of increasing bystander CPR rates [16,17].

The second approach, targeted CPR training, involves spouses and other family members of cardiac arrest victims, the people most likely to witness the event [18-20]. Many authors have suggested targeting family members of patients with known cardiovascular disease for CPR training [21-32]. As few as 9% of this target group have generally received CPR training, while the highest rate is found in Detroit at 47.4% [20,33-36]. Although one study associated advancing age with failing to succeed in CPR training [37], other publications show a high success rate in the older population group [38-40]. While some authors suggest the addition of counselling to deal with the extra responsibility and stress associated with being a potential CPR provider [41,42], CPR training was shown to decrease anxiety, and increase emotional adjustment in family members of cardiac arrest survivors [43-45]. Since 40% of cardiac arrest victims never had any documented cardiovascular disease preceding the event [4], targeting only family members of patients with known cardiovascular disease may not achieve the desired goal. Instead, we should find ways to target the whole population at risk, namely the male and female population aged 55 and older.

Irrespective of the teaching method, retention of CPR knowledge and skills is poor [46-48]. The Heart and Stroke Foundation of Canada currently recommends yearly retraining [49]. There is a large amount of literature demonstrating a significant decrease in CPR knowledge and skills after one year [50-62]. Trainees may go back to pre-training skill levels as early as six months after their CPR class [63-67]. Some evidence exists that re-training may be protective against a decline in CPR skills [68].

### Barriers to CPR training

Authors have asked many people why they were not interested in taking a CPR class. A common response from lay persons is that it simply never occurred to them that CPR training was something they should be doing. Interest in CPR training appears to decrease with advancing age [69-73]. When a group of elders were asked why they had not sought CPR training in the past, most gave no specific reason, or mentioned the inconvenience of having to leave the house, bad health, or cost [74,75]. Some respondents did not understand why they should perform CPR when they can call 9-1-1 [76]. Other common reasons for not seeking CPR training were lack of time or interest, inability to find a course, physical limitations, fear of contracting HIV, or fear of being sued despite the absence of any successful lawsuit for having provided CPR [77,78]. To the contrary, the notion of negligence and failure to provide support could theoretically have legal consequences [79]. There is a lack of consistency among what various studies have found, and none of them clearly demonstrated which factors were most susceptible to influence someone's decision to seek CPR training.

It is also important to address the fear of contracting an infectious disease. Historically, tuberculosis and polio were major concerns on the minds of potential rescuers [80]. In our time, HIV and hepatitis B have replaced those diseases [80,81]. Two surveys published in the late eighties reported that up to 50% of CPR students and instructors alike believed HIV to be transmissible despite standard precautions or simply from giving mouth-to-mouth to a manikin [82,83]. In another survey published during the same period, 90% of homosexuals knew that HIV could not be transmitted from mouth-to-mouth ventilations [84]. No case of HIV, hepatitis B, hepatitis C, or Creutzfeldt-Jakob disease has ever been reported as a result of providing basic CPR to a victim or a manikin [85,86]. There are only three documented cases of horizontal transmission of HIV to health care professionals and they all involved a significant amount of blood and the absence of basic precautions [85]. In a time where people are better educated about the transmission of communicable diseases, we need to re-examine what effect the fear of HIV has on bystander CPR.

### Barriers to CPR performance

Besides fear of disease transmission, there exist other deterrents to bystander CPR. Mouth-to-mouth ventilation is an intimate act that may influence the decision of potential rescuers to perform CPR [87,88]. Willingness to perform CPR appears directly related to the closeness of the relationship with the victim [13,25,34,75,89-92]. In a survey conducted in 1995, 68% mentioned they would perform only chest compressions on a stranger [93]. Other conditions such as vomit, dentures, blood, body odour, and alcohol smell may be unexpected, and have been associated with reluctance to provide CPR to a cardiac arrest victim [94-97]. Nobody knows what impact these conditions have on bystander CPR rates.

CPR certification is associated with increased confidence in one's ability to provide care, which in turn is associated with an increased helping behaviour [27,33,98-101]. But CPR training is still not an assurance of action when the time comes for the rescuer to apply what he or she has learned [102]. Social scientists have studied the effects of ambiguity and the presence of other bystanders on helping behaviour. The more obvious it is that actions are required in a specific situation, the more likely it is that someone will help [103-106]. It has been suggested that people are often unable to make the decision to help rather than choosing not to help [104]. Furthermore, it has been demonstrated that overall helping behaviour decreases with an increasing number of bystanders, a phenomenon known as diffusion of responsibility [106-110]. Although the likelihood of finding someone with CPR training in a crowd increases, the helping behaviour of that rescuer will be reduced by the presence of the other bystanders. Simple and complex behavioural methods have been postulated to address those issues, most of them involving one form or the other of behavioural or cognitive approaches to teaching helping behaviour, but none specifically during CPR classes [111-113]. Once again, we do not fully understand what truly motivates an individual to perform CPR or not when it is necessary to do so.

### Theory of Planned Behaviour

While it remains unclear from previous research if situation-specific factors such as the ones mentioned previously affect the likelihood of CPR-related behaviours, it may also be that general constructs known to affect other health-related behaviours are also relevant. Examination of CPR training and performance behaviours have never been studied in the context of these more general theories, despite their utility in explaining a wide variety of health behaviours [114-118]. For example, the theory of planned behaviour (TPB) proposes that the strength of an individual's intention (or motivation) to engage in a behaviour, and the degree of control they feel they have over that behaviour (perceived behavioural control) are the proximal determinants of engaging in it [119]. The TPB also proposes that intention strength is determined by three variables: attitudes towards the behaviour (a product of beliefs about its consequences and evaluations of those consequences), subjective norms (a product of perceptions of the views of other individuals or groups about the behaviour, and the strength of the individual's desire to gain approval of these groups) and perceived behavioural control (a function of beliefs about factors likely to facilitate or inhibit the behaviour – these might include organizational constraints and patient preferences). The TPB has been shown to predict a range of individual health related behaviours with some success [120,121] (e.g. recent meta-analyses have suggested that the TPB can account for around 20% of the variance in health behaviour [122-124]). To our knowledge, the TPB has never been utilized to study bystander's motivations with regard to CPR training and performance. However, early studies suggest that it is a useful, systematic tool to identify barriers to and facilitators of change and hence appropriate forms of intervention [122-128].

### Systematic review of the literature on bystander CPR determinants

CPR is a crucial yet weak link of the Chain of Survival for out-of-hospital cardiac arrest. We sought to understand what is known about the determinants of bystander CPR and factors associated with successful CPR training by conducting a systematic review of the available literature [129]. Eleven electronic databases, one trial registry, and nine scientific web sites were searched. In addition, hand searches were performed and six content experts were contacted. All communications pertaining to WHO should learn CPR, WHAT should be taught, WHEN to repeat training, WHERE to give CPR instructions, and WHY people lack the motivation to learn and perform CPR were reviewed with restriction. Publications were grouped by category and recommendations were developed using a standardized classification system based on level of evidence.

A total of 252 articles were included out of 2,409 located. Differences in their study design precluded a meta-analysis. Twenty-two recommendations were classified. Recommendations with the highest scores (A, I-2) were: 1) Dispatch-assisted CPR instructions; 2) Teaching CPR to family members of cardiac patients; 3) Braslow's self-training video; 4) Maximizing time spent using manikins; and 5) Teaching concepts of ambiguity and diffusion of responsibility. Other examples include: Mass training events (C, II-3), pulse taking by laymen (D, I-2), and CPR using chest compressions alone (E, I-2).

This exercise resulted in the evaluation and classification of the potential impact of interventions designed to improve bystander CPR rates. These results will help identify common barriers and facilitators to CPR training and CPR performance for the purpose of the current project, more specifically for the purpose of including theoretical constructs represented in the literature.

## Objectives

The overall goal of this study is to design and conduct a survey to better understand the behavioural factors associated with successful CPR knowledge transfer in independent-living older Canadians (aged 55 or older).

Specific objectives are:

1) To conduct semi-structured qualitative interviews to identify factors influencing CPR training and performance behaviours;

2) To develop a survey instrument about factors influencing CPR training and performance behaviours based on a systematic review of the literature [129], the results of the semi-structured interviews, and theoretical constructs from the Theory of Planned Behaviour;

3) To conduct a telephone survey among an independent-living population aged 55 or more using the survey instrument, and to identify factors and strategies that might be targeted by knowledge translation interventions.

## Methods/Design

### Study design

A multi-phase approach will be taken to develop, pilot-test, and administer a telephone survey examining the determinants of CPR training and performance among a population aged 55 or older. In the first phase, data will be used from a recently completed systematic review of the literature [129] to inform the development of a semi-structured interview guide designed to identify barriers and facilitators to a) obtaining CPR training, and b) actually performing CPR, among our target population. Interviews of people aged 55 and older will be carried out until information saturation is achieved; we estimate no more than 20 interviews will be necessary. In the second phase, relevant barriers/facilitators to the two CPR-related behaviours from the interviews will inform development of a pilot telephone survey using the theoretical framework provided by the Theory of Planned Behaviour. In the third phase, a telephone survey will be conducted on individuals over the age of 55 to identify which combinations of barriers, facilitators, and theoretical constructs best predict intention to receive CPR training and/or to perform CPR on a cardiac arrest victim.

### Method of assessment and data collection

#### Phase one – semi-structured interviews

The purpose of this phase of the research is to identify and describe barriers and facilitators about CPR training and CPR performance among our target population, and examine this group's perceptions of the factors influencing their behaviour. This will be accomplished by conducting semi-structured interviews on a convenience sample of people from the target population. Data from this preliminary work will be used to describe all relevant barriers/facilitators to these two target behaviours in this high impact population, and inform the content of the larger scale telephone survey.

An interview guide will be developed using the results of the systematic review [129]. A research assistant will be trained in interviewing skills and will conduct the interviews either in person or over the telephone.

Approximately 20 individuals (male and female) from the urban and sub-urban Ottawa region will be interviewed. While 6–8 participants are often enough for a homogeneous sample, 12–20 are commonly needed when looking for disconfirming evidence or trying to achieve maximum variation [130-132].

The interviews will yield a large quantity of data. To monitor the progress of the interviews and permit follow-up of issues that may emerge from the data, interviewing, transcription, and analysis will proceed concurrently. The audio-tapes will be transcribed verbatim and verified by the interviewer prior to analysis. Data will be imported into a qualitative software package (Nu Dist NVivo™) to facilitate thematic coding, evaluation and analysis [133]. The analysis will be consistent with the methods used for thematic analysis and thick description [130,132,134,135].

#### Phase two – survey devlopement

The purpose of this phase is to use the data generated from the interviews to develop and pilot test a psychometrically sound telephone survey instrument examining issues around our two target behaviours in the context of a well-validated theoretical framework.

Based on now-standard methodologies for developing context-specific measures of the general constructs proposed in the Theory of Planned Behaviour [124,125,136-138], a content analysis of the qualitative data generated from the interviews (by at least two researchers) will identify: 1) the most frequently perceived advantages and disadvantages of performing the behaviour (behavioural beliefs); 2) the most important people or groups of people who would approve or disapprove of one's performance of this behaviour (normative beliefs); and 3) the list of the perceived barriers or facilitating factors that could hamper or facilitate adoption of the behaviour studied (control beliefs). The two behaviours under study will be "seeking CPR training" and "providing CPR to a cardiac arrest victim". The survey will be organized using the theoretical constructs of the Theory of Planned Behaviour which measure: behavioural intentions, attitudes, subjective norms, and perceived behavioural control. Assessments for each of the four theoretical constructs will include direct and indirect belief-based measures; each measure will use a minimum of three items on a 7-point Likert scale. The first draft of the questionnaire will be developed using standard question stems and responses to measure beliefs most often listed (usually 75% of all beliefs stated) [136].

An initial draft of the survey will be circulated around the extended project team to ensure face and content validity. We will pilot the survey with 10 participants twice over a two week period for clarity, acceptability, and test-retest reliability. Data from the pilot survey will be analysed for temporal stability and internal consistency using standard techniques and revised if necessary [139].

#### Phase three – survey administration

The purpose of this phase of the research is to survey our target population using the survey instrument developed in the survey development phase.

We intend to recruit a representative sample of people over the age of 55 in the Canadian population. Our respondents will be stratified by province (excluding the territories), and urban (greater than 100,000 population) versus rural communities.

The survey will be administered using random digit dialling. The administration protocol will include a pre-test of 10 interviews, a minimum of 12 calls/attempts to each random telephone number to obtain a call outcome, and one attempt to convert all first refusals. Ten percent of all interviewers' work will be monitored for quality assurance purposes. We anticipate a response rate of at least 60%.

### Data analysis

The primary hypotheses of the third phase of the project (Survey Phase) will involve examining whether constructs comprising the Theory of Planned Behaviour are significantly related to our two primary outcomes. Specifically:

Hypothesis 1: Attitudes, subjective norms, and perceived behavioural control will be significant predictors of intention to engage in CPR training, and

Hypothesis 2: Attitudes, subjective norms, and perceived behavioural control will be significant predictors of intention to engage in intention to perform CPR in appropriate circumstances.

Analysis of the survey data will first involve descriptive statistics describing the most commonly cited barriers/facilitators to our two target behaviors. Analysis of Hypothesis 1 and 2 will be carried out by multiple regression. For each of the two target behaviours (intention to engage in CPR training, intention to perform CPR), a blocked multiple regression will test the strength of relationship of these outcomes with the constructs predicted by the Theory of Planned Behaviour; i.e. the constructs of attitude, subjective norms, and behavioural control will be added into the model as a block, and any additional constructs suggested by the Interview Phase added to the model to see if it contributes to the model over and above the factors discussed in the Theory of Planned Behaviour.

In addition to standard regression modelling, we will also produce a structural equation model of our data. Structural equation modelling (SEM) allows the researcher to model not only direct relationships between constructs and outcomes (e.g. is perceived behavioural control related to actual behaviour?), but also indirect relationships through intervening constructs (e.g. is the effect of perceived behavioural control on intention mediated by other constructs?). Because the constructs in the structural model are determined theoretically rather than statistically, this technique is useful for testing the validity of whole theories within a single analysis. Furthermore, SEM does not assume perfect measurement of the constructs, as does regression, but rather explicitly measures and excludes error in the measurement variables from the definition of the constructs.

### Sample Size

Power calculations for multiple regression analysis depend on the number of cases per predictor variable. A minimum sample size of 50 + 8 m, where m is the number of predictor variables, is recommended for testing the multiple correlation, and 100 + 8 m for testing individual predictors [140,141]. The two behaviours under study will be "seeking CPR training and providing CPR to a cardiac arrest victim". The survey will be organized using the theoretical constructs of the Theory of Planned Behaviour which measure: behavioural intentions, attitudes, subjective norms, and perceived behavioural control. Assessments for each of the four theoretical constructs for both behaviours under study will include direct and indirect belief-based measures; each measure will use a minimum of three items on a 7-point Likert scale. Our survey will require a minimum of 146 respondents (assuming three confounders per theoretical construct). We will over sample to handle incomplete data, and target 200 participants.

### Ethics approval

This study has been approved by the Research Ethics Board of the Ottawa Hospital (protocol # 2007751-01H).

## Discussion

The proposed survey will address important knowledge gaps in our understanding of the barriers and facilitators of CPR training and CPR performance. Several attempts have been made to elucidate these behaviours in the past. For the most part, these consisted in poorly developed and constructed surveys, with no follow-up intervention based on their findings.

The results of the proposed survey, based on scientifically developed theoretical constructs, will provide invaluable insight into factors influencing the interest in CPR training and performance among a targeted group of individuals most susceptible to witnessing a victim in cardiac arrest. We plan to apply our findings and evaluate their impact during randomized controlled trials of various interventions designed to promote attendance at CPR classes., and CPR performance. The results of this survey should contribute to the development of the updated version of the *Guidelines for Resuscitation *in 2010, and offer scientific evidence to support a paradigm shift in the approach to CPR training in the community. Ultimately, the most reliable measure of impact from all proposed interventions will be an unequivocal increase in bystander and survival rates for out-of-hospital cardiac arrest victims.

## Competing interests

The authors declare that they have no competing interests.

## Authors' contributions

CV conceived of the study and drafted the manuscript. JG, JB and IS assisted with the methodology and revised it critically for important intellectual content. GW assisted with the statistical design and methodology. MC helped draft and edit the manuscript. All authors read and approved the final manuscript.

## Pre-publication history

The pre-publication history for this paper can be accessed here:


